# Complete Genome Sequence of *Bradyrhizobium* sp. Strain CCGE-LA001, Isolated from Field Nodules of the Enigmatic Wild Bean *Phaseolus microcarpus*

**DOI:** 10.1128/genomeA.00126-16

**Published:** 2016-03-17

**Authors:** Luis E. Servín-Garcidueñas, Marco A. Rogel, Ernesto Ormeño-Orrillo, Alejandra Zayas-del Moral, Federico Sánchez, Esperanza Martínez-Romero

**Affiliations:** a Centro de Ciencias de la Complejidad, UNAM, Ciudad de México, México; b Laboratorio Nacional de Análisis y Síntesis Ecológica para la Conservación de Recursos Genéticos, Escuela Nacional de Estudios Superiores Unidad Morelia, UNAM, Morelia, Michoacán, México; c Centro de Ciencias Genómicas, UNAM, Cuernavaca, Morelos, México; d Universidad Nacional Agraria La Molina, Lima, Perú; e Instituto de Biotecnología, UNAM, Cuernavaca, Morelos, México

## Abstract

We present the complete genome sequence of *Bradyrhizobium* sp. strain CCGE-LA001, a nitrogen-fixing bacterium isolated from nodules of *Phaseolus microcarpus.* Strain CCGE-LA001 represents the first sequenced bradyrhizobial strain obtained from a wild *Phaseolus* sp. Its genome revealed a large and novel symbiotic island.

## GENOME ANNOUNCEMENT

*Phaseolus microcarpus* Mart (1831) is a wild bean species that grows as an annual vine with small pods containing one or two 4- to 6-mm-wide parallelepiped seeds*.* Currently*, P. microcarpus* is considered an enigmatic species with a weakly resolved phylogeny (1, 2). Recently, we isolated *Bradyrhizobium* strains from field-collected *P. microcarpus* nodules from the town of Oaxtepec, Morelos, Mexico. A representative strain designated CCGE-LA001 was sequenced and its draft genome was obtained (3). Here, we present the complete genome sequence of *Bradyrhizobium* sp. strain CCGE-LA001.

The CCGE-LA001 genome was sequenced using the Pacific Biosciences (PacBio RSII) single-molecule real-time (SMRT) sequencing platform. PacBio reads were processed, mapped, and *de novo* assembled by the SMRT analysis pipeline using the HGAP3 protocol and polished using Quiver (4) to give a fully closed genome with 243× coverage. The total genome size was 7,833,499 bp with a G+C content of 63.6% and coded for 7,077 predicted open reading frames. Genome annotation was performed using the NCBI Prokaryotic Genome Annotation Pipeline (http://www.ncbi.nlm.nih.gov/genome/annotation_prok). Average nucleotide identity (ANI) values were calculated as previously proposed (5) using the ANI calculator from the Kostas lab (http://enve-omics.ce.gatech.edu/ani).

The CCGE-LA001 genome contains a 790-kb symbiotic island delimited by methionine tRNA genes. Remarkably, this symbiotic island is the largest detected to date for a bradyrhizobial strain and is not similar to other symbiotic islands previously reported. The symbiotic island carries genes required for nodulation, conjugative transfer, the type IV secretion system, and several mobilization and hypothetical proteins, among others. Moreover, the diverse repertoire of genes required for nodulation and Nod factor decoration were divergent, with around 70% sequence identity with the corresponding sequences of other *Bradyrhizobium* strains (3). Also, putative genes not found in other currently sequenced bradyrhizobial genomes, including heavy-metal resistance and insecticidal toxin proteins, were identified in the chromosome.

A small-subunit rRNA gene phylogeny placed CCGE-LA001 in the neighborhood of *Bradyrhizobium japonicum*, but CCGE-LA001 forms no nodules in soybean. However, CCGE-LA001 is able to nodulate and fix nitrogen in its natural host *P. microcarpus* as well as in *Vigna unguiculata* and *Phaseolus acutifolius*, but, in the latter, nodules were small and not abundant. A concatenated phylogeny using *recA*, *glnII*, *gyrB*, and *dnaK* genes increased the taxonomy resolution and placed CCGE-LA001 in the vicinity of *Bradyrhizobium daqingense*, within a phylogenetic group including other bradyrhizobial type species like *B. yuanmingense*, *B. arachidis*, *B. huanghuaihaiense*, and *B. liaoningense* (3). An ANI boundary of 95 to 96% has been useful for taxonomically circumscribing prokaryotic species (6). Genome comparisons between CCGE-LA001 and other currently sequenced bradyrhizobial strains revealed ANI values well under 88%. However, genome sequences from more phylogenetically related type strains are needed for further comparisons.

The genome from a natural symbiont of *P. microcarpus* will aid in better defining the molecular basis of *Phaseolus* specificity and contribute to the *Bradyrhizobium* pangenome. This genome and other sequenced genomes will be used for comparative genomics analyses of *Bradyrhizobium* strains nodulating *Phaseolus* species.

### Nucleotide sequence accession number.

This whole-genome shotgun project has been deposited in GenBank under the accession number CP013949.
